# Proteomic analysis identifies a signature of disease severity in the plasma of COVID-19 pneumonia patients associated to neutrophil, platelet and complement activation

**DOI:** 10.1186/s12014-022-09377-7

**Published:** 2022-11-08

**Authors:** Fabiola Ciccosanti, Manuela Antonioli, Alessandra Sacchi, Stefania Notari, Anna Farina, Alessia Beccacece, Marisa Fusto, Alessandra Vergori, Gianpiero D’Offizi, Fabrizio Taglietti, Andrea Antinori, Emanuele Nicastri, Luisa Marchioni, Fabrizio Palmieri, Giuseppe Ippolito, Mauro Piacentini, Chiara Agrati, Gian Maria Fimia

**Affiliations:** 1 Department of Epidemiology, Preclinical Research and Advanced Diagnostics, National Institute for Infectious Diseases IRCCS “L. Spallanzani”, Rome, Italy; 2 Infectious Disease-Clinical Department, National Institute for Infectious Diseases IRCCS “L. Spallanzani”, Rome, Italy; 3 Department of Biology, University of Rome “Tor Vergata”, Rome, Italy; 4 Department of Molecular Medicine, University of Rome “Sapienza”, Rome, Italy; 5 Present Address: General Directorate for Research and Health Innovation, Italian Ministry of Health, Rome, Italy; 6 Present Address: Department of Hematology/Oncology and Cell and Gene Therapy, Bambino Gesù Children Hospital, IRCCS, Rome, Italy

**Keywords:** SARS CoV 2, Proteomics, Plasma, Complement, Neutrophils, Platelets

## Abstract

Most patients infected with SARS-CoV-2 display mild symptoms with good prognosis, while 20% of patients suffer from severe viral pneumonia and up to 5% may require intensive care unit (ICU) admission due to severe acute respiratory syndrome, which could be accompanied by multiorgan failure.

Plasma proteomics provide valuable and unbiased information about disease progression and therapeutic candidates. Recent proteomic studies have identified molecular changes in plasma of COVID-19 patients that implied significant dysregulation of several aspects of the inflammatory response accompanied by a general metabolic suppression. However, which of these plasma alterations are associated with disease severity remains only partly characterized.

A known limitation of proteomic studies of plasma samples is the large difference in the macromolecule abundance, with concentration spanning at least 10 orders of magnitude. To improve the coverage of plasma contents, we performed a deep proteomic analysis of plasma from 10 COVID-19 patients with severe/fatal pneumonia compared to 10 COVID-19 patients with pneumonia who did not require ICU admission (non-ICU). To this aim, plasma samples were first depleted of the most abundant proteins, trypsin digested and peptides subjected to a high pH reversed-phase peptide fractionation before LC–MS analysis.

These results highlighted an increase of proteins involved in neutrophil and platelet activity and acute phase response, which is significantly higher in severe/fatal COVID-19 patients when compared to non-ICU ones. Importantly, these changes are associated with a selective induction of complement cascade factors in severe/fatal COVID-19 patients. Data are available via ProteomeXchange with identifier PXD036491. Among these alterations, we confirmed by ELISA that higher levels of the neutrophil granule proteins DEFA3 and LCN2 are present in COVID-19 patients requiring ICU admission when compared to non-ICU and healthy donors.

Altogether, our study provided an in-depth view of plasma proteome changes that occur in COVID-19 patients in relation to disease severity, which can be helpful to identify therapeutic strategies to improve the disease outcome.

**Supplementary Information:**

The online version contains supplementary material available at 10.1186/s12014-022-09377-7.

## Introduction

SARS-CoV-2 infection promotes the development of COVID-19 diseasewhich is characterized by a wide spectrum of clinical symptomsranging from an asymptomatic state to a life-threatening infection [1]. In many cases the prognosis is favorablebut about 20% of patients suffer from respiratory distressrequiring different types of oxygen therapy up to mechanical ventilationwhich can be accompanied by multiorgan failure [2]. Such patients also may develop neurological problems or hematological abnormalities and suffer venous thromboembolism [3]. The mortality rate is higher among the elderly or people with chronic diseases [4, 5]. Various comorbidities contribute to worsen the prognosis in COVID-19 patientsincluding obesitydiabetescardiovascular diseaseand immunosuppression [6–8].

While the disease etiology of COVID-19 is progressively being unraveledthe underlying molecular mechanisms and the associated metabolic alterations remain less understood [9, 10]. This is also due to the fact thatin addition to viral factorsdisease severity appears to depend on host factorssupporting the need to better understand the signature of individuals’ responses at a molecular level [11]. For examplethe molecular changes observed in COVID-19 patients’ sera have highlighted a complex dysregulation of platelet and neutrophil degranulationmacrophage function and the complement system [12–16]. Neutrophil dysregulation is associated with COVID-19 severity [17]. Moreovera rapid decline of lung function observed in patients admitted to intensive care units is associated with a dysregulated immune responsedefined “cytokine storm”characterized by increased circulating levels of pro-inflammatory interleukins [18]. The systemic hyperinflammation characterizing severe COVID-19 was associated with different alterations of immune responseincluding lymphopeniamassive bystander T cell activationT cell exhaustion and cytotoxic lymphocyte impairment [19–21]. In contrastin SARS-CoV-2 patients experiencing mild disease a balanced and well-orchestrated immune response was described [22].

Ventilator support is often insufficient for preventing mortality in the most severe casesdue to the presence of widespread thrombotic microangiopathywhich results in multiorgan failure and death [23, 24].

In this scenariothere is therefore an urgent need for more in-depth characterization of COVID-19 induced alterations of host pathways and how they are functionally interrelatedwith the aim of better deciphering the disease’s complexity by identifying additional factors that can help to interpret the patient’s response to SARS-CoV-2 infection.

Changes in human plasma protein levels have been well recognized as indicators of pathophysiological changes associated with various disease statesincluding viral infections. Howevera known limitation of proteomic studies of plasma samples is the large difference in the macromolecule abundancewith concentration spanning at least 10 orders of magnitude [25]. To improve the coverage of plasma contents our study undertook an in-depth plasma analysis in COVID-19 patients requiring ICU admission by combining the depletion of most abundant proteins with a high pH reversed-phase peptide fractionation before LC–MS analysis.

## Materials and methods

### Patient selection

This study was approved by the National Institute for Infectious Diseases L. Spallanzani IRCCS Ethics CommitteeRomeItaly. Written informed consent for plasma donation was obtained from all patients and healthy donors (HDs). SARS-CoV-2 infected patients (n = 32) were diagnosed with COVID-19 by SARS-CoV-2 RT-PCR performed on nasopharyngeal and oropharyngeal swabs. We enrolled SARS-CoV-2 positive patientsall with pneumoniarevealed with high resolution chest computed tomography, without other infections such as HCV, HBV, MTB, and others; one patient in each group tested by mass spectrometry was HIV positive. Plasma samples were collected in the first few days of clinical hospitalization. Partial pressure of oxygen/inspired oxygen concentration ratio (PaO2/FiO2 ratio) which represents a valuable clinical measure of the patient’s respiratory status for the evaluation of assisted oxygenationwere recorded during the hospitalization. Demographicsclinical baseline characteristicstherapy and cause of death are shown in Additional file 1: Table S1. For the HDs control groupplasma donation to our biobank was obtained in the pre-COVID-19 time.

### Plasma separation from peripheral blood sampling

Peripheral blood in K2-Ethylenediaminetetraacetic acid (EDTA) BD Vacutainer® blood collection tubes (BD Biosciences Franklin LakesNJUSA) was centrifuged at 1400 RPM for 10 min to obtain the plasma and aliquoted and stored at − 80 °C until use.

### Plasma protein purification peptide purification and LC–MS/MS analysis

To remove high abundance proteins,100ul of plasma for each samplepre-treated with Triton-X100 (Merck Life Science)at a final concentration of 1%to make the samples safewere processed with High-select Top14 Abundant Protein Depletion Resin columns (Thermo Fisher Scientific) following the manufacturer’s protocol. The depleted samples were eluted in 10 mM PBS and 0.02% sodium azidepH 7,4.

From each sample 14ug of purified proteins were boiled at 95 °C and treated with DL-dithiothreitol (10 mM at 56CMerck Life Science) and iodoacetamide (55 mM at room temperatureMerck Life Science) for disulfide bond reduction and alkylation respectively. Then the samples were diluted 10 times by adding 50 mM NH4HCO3 (Merck Life Science) 1 M urea (Merck Life Science) and digested with trypsin (0.4 μg/samplePromega) at 37 °C overnight.

The peptide mixture was acidified with trifluoroacetic acid (TFAfinal concentration 0.1%Fluka) and fractionatedbased on the peptides hydrophobicityusing Pierce High pH Reverse Phase Peptide Fractionation Kit (Thermo Fisher Scientific) following the manufacturer's protocol.

Peptides in each fraction (8/sample) were driedresuspended in 2.5% acetonitrile (ACNFluka)0.1% TFA and 0.1% formic acid (Fluka) and then analyzed twiceas technical replicatesby ultra-high performance liquid chromatography coupled with high resolution mass spectrometry using Thermo Scientific Q Exactive Plus Orbitrap as described [26]. In particularthe peptides were separated by nano liquid chromatography (UltiMate 3000 RSLC nano-LC systemThermo Fisher Scientific)loaded onto a 75 μm C18 column (ES800-PepMap™ RSLC C18150 mm × 75 μmThermo Fisher Scientific)using a 100 min linear multistep gradient elution (from 4 to 90% eluent B80% ACN0.1% formic acidwith a constant flow rate of 0.3 μL/min)and were analyzed by Q Exactive plus™ Hybrid Quadrupole-Orbitrap™ Mass Spectrometer (Thermo Fisher Scientific) with this run condition: electrospray ionization (ESI) voltage 2.0 kV and MS data were acquired in a positive mode using data-dependent mode selecting the 15 most intense ions with full scan MS spectra range from m/z 350.0 to m/z 1700.0resolution of 70,000injection time 100 msAGC target 3 × 106isolation window ± 2.0 m/zcharge exclusion 1,7,8 > 8 and the dynamic exclusion 20 s. For HCD fragmentationresolution was set to 17,500AGC target to 10000 and injection time of 80 ms.

### Quantification and statistical analysis

The raw data from the mass spectrometric analysis were processed using the MaxQuant software v.1.5.5.1 and quantified using the iBAQ algorithm [27]. Data were analyzed with Perseus software v.1.6.15 [28] (http://www.perseus-framework.org). Brieflydata were filtered based on categorical rows; only identified by sidereverse and potential contaminants were removed. Calculated iBAQ were log2 convertedgrouped to compare HDnon-ICU and ICUand filtered based on proteins identified in at least one group with a coverage of 90%. Each set was normalized by calculating the median of log2-transformed iBAQ intensities for each MS run and subtracting it from each log2-transformed iBAQ intensity in the same run. Missing values were imputed from normal distribution based on total matrix (width 0.3 and down shift 1.3). The obtained matrix of 216 identifications was used to calculate the Principal Component Analysis (2 componentsFDR < 0.05) and to generate a volcano plot for HD vs COVID-19 non-ICUHD vs COVID-19 ICU/F groups and COVID-19 non-ICU vs COVID-19 ICU/F groups with 250 randomizations based on two sides t-test (FDR < 0.05S0 = 0.1). Hierarchical clustering of identified proteins was performed after Z-score normalization thensignificantly different proteins among groups were calculated by multiple-sample test (ANOVA) and Post hoc Tukey’s HSD test of one-way ANOVA (FDR < 0.05). Euclidean distance were used to compare different groups and clusters were extracted using n = 4 and n = 15 to allow a better discrimination of lower abundant proteins.

Plasma proteins that underwent significant fold changes were considered for the classification of biological dysregulated processes. Only processes relevant for plasma proteins were included in the list. The network analysis and the visualization of plasma proteins significantly modulated in COVID-19 non-ICU and COVID-19 ICU/F was performed using the application of String-DB specifically developed for Cytoscape (v3.8.2) (StringApp) [29]. Functional enrichment analysis was performed for each of identified cluster and significant GO process were revaluated (FDR < 0.05). Graph pad Prism 8 was used to generate reported plots.

### ELISA assay

Plasma protein levels were measured using a Human ELISA kit (MyBioSource) following the manufacturer's protocol. Plasma was diluted 1:9 for the Human MPO kit assay (MBS175830)1:20 for the Human DEFA3 kit assay (MBS2705383) and 1:2 for the Human NGAL kit assay (MBS268158).

Statistical analyses were performed using Kruskal–Wallis one-way analysis of variance (ANOVA) with Dunn’s post hoc analysis. Statistics were carried out using GraphPad Prism 5.00. P-values less than 0.05 were considered significant.

## Results

### Patients and experimental design

**Fig. 1:**
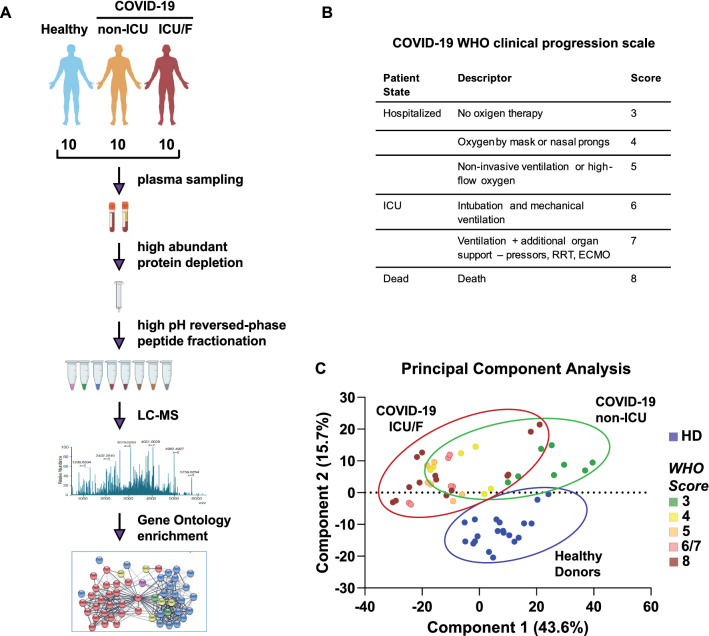
**A** Overview of blood samples collection from COVID-19 patientsincluding 8 patients with severe pneumonia who required ICU admission2 patients who died before ICU admission (F: fatal)10 COVID-19 patients with pneumonia who did not require ICU admissionand 10 Healthy volunteers. The workflow for processing the proteomic data are shownincluding the plasma separationabundant protein depletiontrypsin digestionpeptide fractionationLCMS/MS analysisdatabase searchand further computational analyses. **B** Patients were classified based on the WHO scale from severity ‘‘3’’ (hospitalizedno oxygen therapy) to severity ‘‘6/7’’ (ICU admission) and “8” (death). **C** Principal Component Analysis (PCA) based on the entire dataset of HD and COVID-19 patients. Healthy and COVID-19 patients are well discriminated along the second principle componentwhile PC1 partially discriminate ICU/F and non-ICU COVID-19 patients

Blood samples were collected from a cohort of 20 patients with COVID-19 pneumonia admitted to INMI L. Spallanzani (RomeItaly)including 8 patients with severe pneumonia who required ICU admission (ICU)2 patients who died before ICU admission (F = fatal) and 10 COVID-19 patients with pneumonia who did not require ICU admission (non-ICU) (Fig. 1A). Severity of COVID-19 was graded using the WHO ordinal outcome scale of clinical improvement (Fig. 1B, https://www.who.int/teams/blueprint/covid-19). Among COVID-19 ICU patients5 died and 3 had survived and been discharged from the hospital. Among COVID-19 non-ICU patients6 required non-invasive oxygen therapy (non-invasive ventilation (NIV)/continuous positive airway pressure mask (CPAP) n = 3large-reservoir Venturi masks (Ventimask)/nasal cannula (NC) n = 3) while 4 did not require any of these devices. Most of the COVID-19 patients suffered from at least one chronic disease (ICU/F: 9/10; non-ICU: 6/10) (Additional file 1: Table S1).

Blood samples from 10 healthy donors (HD) collected before SARS-CoV-2 pandemic (before January 2020) were analysed for comparison. Samples were collected early after hospitalization: interquartile range (IQR) 2.5 days for COVID-19 non-ICU (from 1.25–3.75) interquartile range (IQR) 2.75 days for COVID-19 ICU/F (from 2–4.75). In the latter groupsamples were collected either before ICU admission (4 patientsfrom 1 to 6 days) or after ICU admission (4 patientsfrom 0 to 8 days).

To improve the coverage of plasma contents14 highly abundant proteinsincluding albuminIgG and fibrinogenwere depleted by affinity chromatography. Proteins were then digested with trypsin (Fig. 1A). Peptides from each sample were then subjected to high pH reversed-phase chromatography and eluted in eight fractions. Each peptide mixture was analyzed by liquid chromatography with tandem mass spectrometry (LC–MS/MS) using a Q-Exactive Plus instrument.

### Proteomic profiling of plasma from COVID-19 patients

Each sample was run twice as technical replicates. MaxQuant software [27] identified about 6100 peptides in totalwith a number of peptides ranging from 1,426 to 3,807 per patient (Additional file 2: Table S2). Considering only proteins matched by 2 independent peptides449 human proteins were identified in totalwith 194 proteins quantified in all samples (40.2%)indicating a good reproducibility of the proteomic profiling (Additional file 3: Table S3). To ensure data qualityonly 216 proteins mutually quantified in > 90% samples were considered for protein level comparison. For each proteinmultivariate normal imputation (MVNI) was applied to impute the missing values [30].

The principal-component analysis (PCA) of the 30 samples was performed by using raw MS/MS values from 216 proteins with normalized expression values across samples (Fig. 1C). Samples from COVID-19 and HD were unambiguously distinguished. Samples from ICU/F and non-ICU COVID-19 samples were not completely separated. InterestinglyCOVID-19 non-ICU patients that overlap with the COVID-19 ICU/F ones are mainly those who required non-invasive oxygen therapies. On the other handthe COVID-19 ICU patient who clusters closer to the COVID-19 non-ICU ones had a blood sample collected 6 days before entering in ICUsuggesting that main changes in proteomic profile were not yet present.

Prompted by this initial evidence that proteomic plasma alterations may correlate with the disease statuswe investigated more in details which specific pathways account for the classification of COVID-19 cases.

### Proteomic alterations in plasma of severe vs non-severe COVID-19 patients

**Fig. 2:**
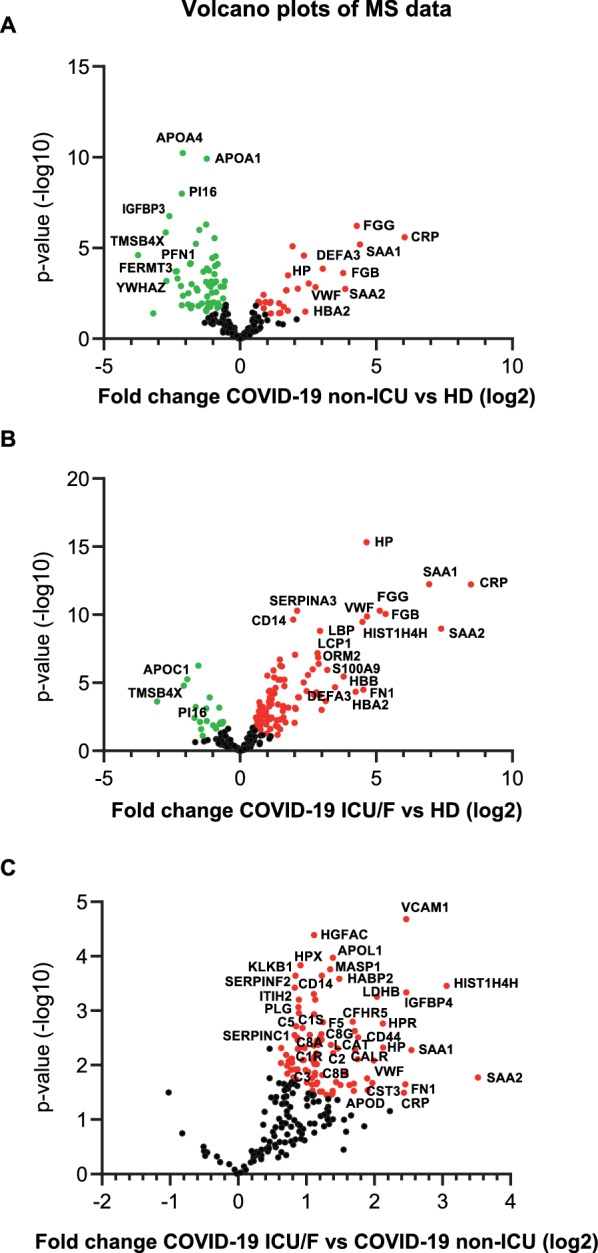
Volcano plots of the − log10 p value vs. the log2 protein abundance comparisons between plasma from control subjects (HD) and those diagnosed with COVID-19 not requiring ICU admission (**A**)requiring ICU admission/fatal cases (**B**) and between non-ICU and ICU/F patients (**C**). Proteins outside the significance threshold lines (adjusted p value < 0.05 and COVID-19/Control > 2 or < 1/2) were colored in red (upregulated) or green (downregulated)

Signatures of COVID-19 were identified by analyzing plasma proteins that underwent significant fold changes (FCs) among different groups (log2 (FC) > 0.5; unpaired two-sided Welch’s t test; p < 0.05). A total of 120 differentially expressed proteins (DEPs) were identified in COVID-19 ICU/F vs HD91 DEPs in COVID-19 non-ICU vs HD and 98 DEPs in COVID-19 ICU/F vs non-ICU (Fig. 2; Additional file 4: Table S4). InterestinglyDEPs that discriminate COVID-19 ICU/F from non-ICU were all upregulated in the first group (Fig. 2C) suggesting that the alterations of plasma proteins became more extensive in compromised conditions.

**Fig. 3:**
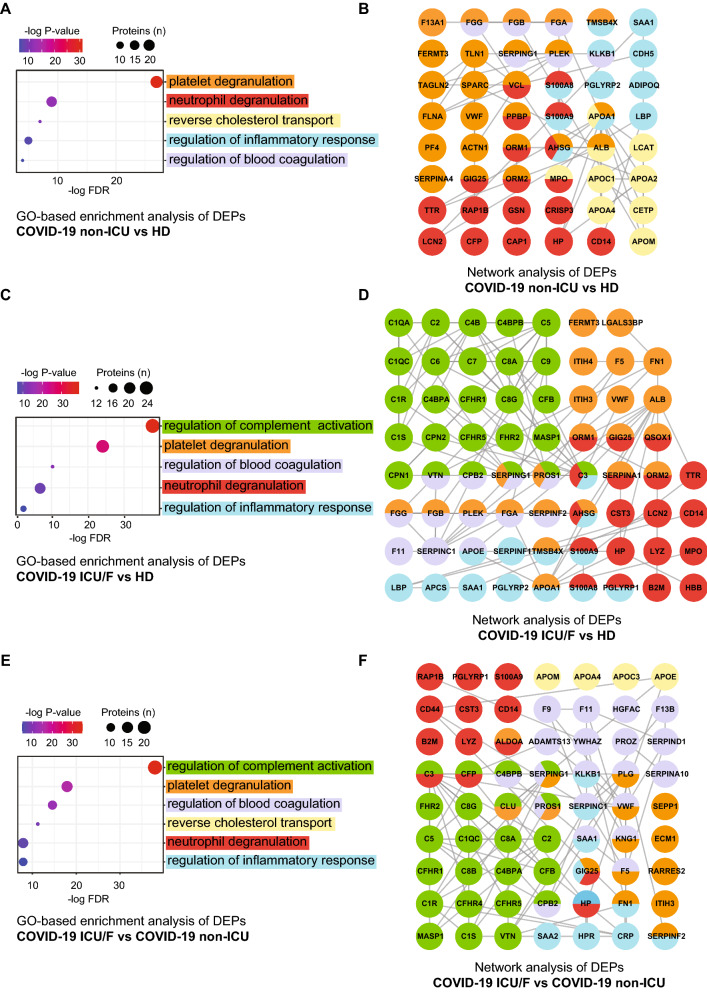
Left panels: GO-based enrichment analysis of DEPs between HD and COVID-19 non-ICU (**A**), HD and COVID-19 ICU/F (**C**), COVID-19 non-ICU and COVID-19 ICU/F (**E**) shown in the term of biological processes (two-sided hypergeometric test; p < 0.001) and the number of counts (n > 10). Redundant GO terms and those not relevant for plasma proteins were omitted (see Additional file 5: Table S5 for the full list). GO terms were sorted by P-Value**.** Right panels: Networks analysis of plasma proteins whose levels changed between HD and COVID-19 non-ICU patients (**B**) HD and COVID-19 ICU/F (**D**), COVID-19 non-ICU and COVID-19 ICU/F (**F**). The colors of the circles indicate the processes in which these proteins play a role as described in ACand E. Edges transparency is based on STRINGdb score

The DEPs were then subjected to Gene Ontology (GO) [31] enrichment and network analyses. In all comparisonsthe GO terms highly enriched in processes are related to inflammationplatelet and neutrophil degranulationand coagulation cascades (Fig. 3A, C and Additional file 5: Table S5). Proteins involved in lipoprotein remodeling are mainly observed in COVID-19 non-ICU patientswhileimportantlycomplement activation become relevant in the COVID-19 ICU patients when compared to both COVID-19 non-ICU and HDsuggesting that dysregulation of this process may account for the more severe forms of COVID-19 leading to ICU and/or death. Although all processes are strictly interrelatedas indicated in the STRING mapsa dense network of interactions is observed between inflammation and neutrophil factorsas well as between coagulation and platelet factors (Fig. 3B and D), which further overlaps with complement proteins in COVID-19 ICU patients (Fig. 3D and F).

### Cluster analysis of biological processes dysregulated in severe vs non-severe COVID-19 patients

**Fig. 4:**
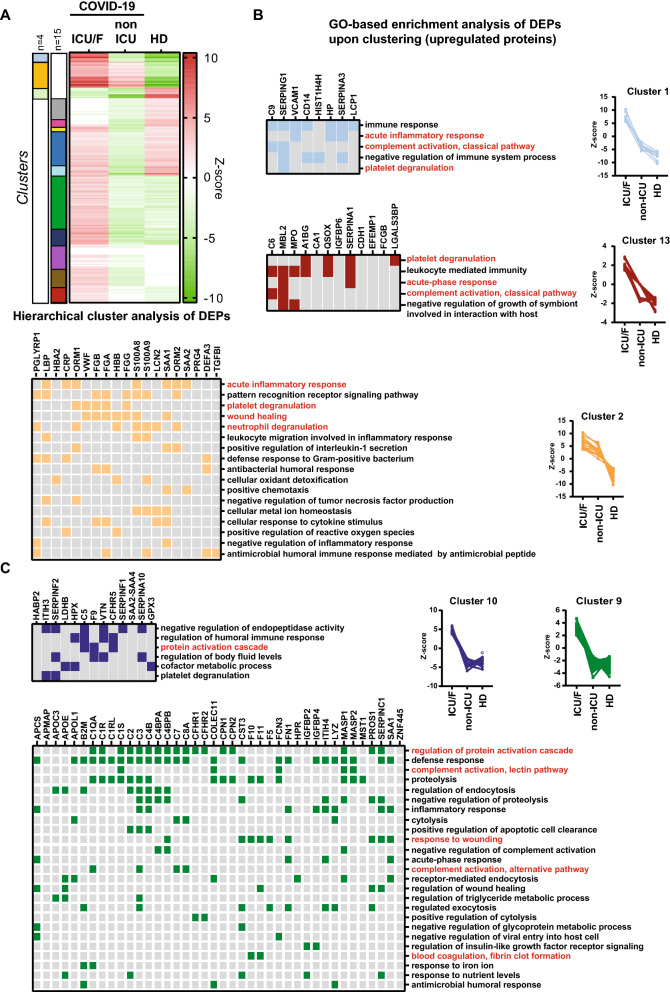
**A** Hierarchical cluster analysis of plasma protein correlation values. Euclidean distance were used to compare different groups and clusters were extracted using either n = 4 or n = 15 to allow a better discrimination of clusters containing lower abundant proteins. **B** GO pathways enriched among plasma proteins that exhibited progressively increased expression in non-ICU and ICU/F COVID-19 patient groups with respect to HDanalysed in each cluster identified in A. **C** GO pathways enriched among plasma proteins that exhibited increased expression mainly in COVID-19 ICU/F patient groups with respect to COVID-19 non-ICU and HDanalysed in each cluster identified in A. Top score processes resulting from the GO-based enrichment analysis (see Fig. 3) are highlighted in red

Since a complex combination of dysregulated processes contribute to the severe forms of COVID-19we evaluated which pathways are concomitantly altered when COVID-19 ICU/F versus COVID-19 non-ICU plasma proteomes were compared by a hierarchical clustering method and results were visualized in a heat map (Fig. 4A). Thirteen different clusters were identified by using two thresholds (n = 4 and n = 15) which were applied to better separate groups either with high or low levels of variations. These clusters were then analysed in relation to the processes to which these proteins play a role (Additional file 6: Table S6).

Cluster 12 and 13 report proteins that are increased in all COVID-19 patients but at higher levels in ICU/F patients (Fig. 4B) which are mainly constituted by proteins playing a role in acute inflammatory response (CRP, ORM1, ORM2, SAA1, SAA2, S100A8, S100A9, Serpin A3). Other highly represented classes are platelet and neutrophil activity (Fig. 4B). In particularhigh levels of HIST1H4H and MPO are associated to Neutrophil extracellular traps (NETs) formationwhile LCN2, PGLYRP1, DEFA3 are neutrophil granule proteins with antimicrobial activity. Importantly, LCN2 were previously reported to be one of the genes predictor for admission to intensive care [17]. Moreoverwe detected the pattern recognition receptor CD14which is expressed by both monocytes and neutrophils and has been proposed as potential target to reduce acute inflammation in COVID-19 patients [32] together with LBP (LPS binding protein) a protein responsible for the potentiation of LPS-mediated activation of monocytes/neutrophils via CD14 [33].

With regard to the platelet activationplatelet granule proteins were detectedsuch as FGA, FGB, FGG, VWF, A1BG and QSOX1, together with LGALS3BP, a multifunctional secreted glycoprotein with prothrombotic and proinflammatory properties [34].

These clusters include only a few members of the complement pathway: C6C9Serpin A1 and G1and MBL2 (Mannose-binding lectin 2) a pattern recognition molecule that initiates the lectin pathway of complement activationwith polymorphisms associated with COVID-19 severity [35].

Various components of the extracellular matrix (ECM) and plasma membrane proteins were also detectedincluding the vascular adhesion protein VCAM1, the platelet adhesion CADHERIN-13the PROTEOGLYCAN 4a protein with anti-inflammatory properties increased in chronic obstructive pulmonary disease [36] TGFBIan ECM factor required for normal alveolar structure and function [37] and EFEMP1 (EGF-containing fibulin-like extracellular matrix protein 1)whose gene is mutated in a macular degeneration disease characterized by complement-trigger protein/lipid deposits [38]. Increased extracellular matrix proteins were previously associated with pulmonary fibrosis [39], with TGFBI having potential roles in extracellular matrix remodeling [40], including lung and cardiac fibrosis [41, 42]. Moreoverthe increase in the hemoglobin proteins HBA1 and HBBthe hemoglobin binding protein (HP) and Carbonic Anhydrase 1 (CA1) both likely indicative of hemolysisas previously reported [43].

Cluster 9 and 10 show proteins that are significantly upregulated only in COVID-19 ICU/F patients (Fig. 4C). The majority of them play a role in the complement cascadeincluding proteins involved in the induction of the different activation pathways (classical pathwaye.g. C1Q, C1R, C1S; alternative pathway, e.g. C3, CFRH1, CFRH2; lectin pathwaye.g. COLEC11, MASP1, MASP2 and FCN3). An increase was also detected for proteins involved in coagulation (F5, F9, F10, F11, PROS1, SERPIN C1, SERPIN A10, SERPIN F2, HABP2) inflammation-induced proteins involved in different types of amyloid diseases (SAA1, SAA2-SAA4, APCS, APMAP, B2M, CST3) and extracellular matrix organization (FN1, VTN, ITH3, ITH4). In additionwe detected an increase in VLDL/LDL lipoprotein (APOC3, APOE, APOL1) andinterestinglyin two multifunctional proteinsGPX3 and MST1/MSPboth playing a role in the regulation of inflammation and lipid metabolism [44, 45].

**Fig. 5:**
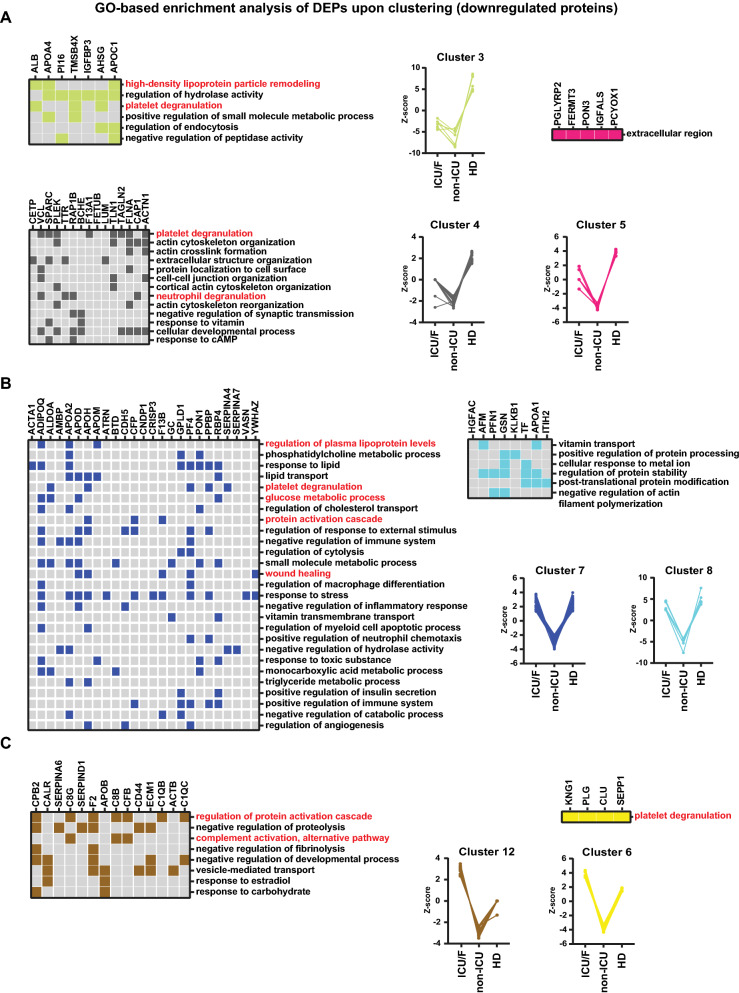
**A** GO pathways enriched among plasma proteins that exhibited decreased expression in both non-ICU and ICU/F COVID-19 patient groups with respect to HDanalysed in each cluster identified in Fig. 4A. **B** GO pathways enriched among plasma proteins that exhibited decreased expression mainly COVID-19 non-ICU patient groups with respect to HD and COVID-19 ICU/F patientsanalysed in each cluster identified in Fig. 4A. **C** GO pathways enriched by plasma proteins that exhibited decreased expression in COVID-19 non-ICU and increased expression in COVID-19 ICU/F patients groups with respect to HDanalysed in each cluster identified in Fig. 4A. Top score processes obtained by the GO-based enrichment analysis (Fig. 3) are highlighted in red

The clusters reporting proteins that are decreased in COVID19 patients include mainly proteins involved in lipid transport and platelet components (Fig. 5). In particularwe observed a decrease in the HDL components APOA4 and APOC1 in both COVID-19 non-ICU and ICU/F patients (cluster 3, Fig. 5A) while APOA2, APOD, APOH, APOM and the HDL-associated PON1 protein appear to be decreased mainly in COVID-19 non-ICU patients (cluster 7, Fig. 5B). Related to lipid metabolismwe also observed a reduction in CETP (cluster 4, Fig. 5A) which is involved in cholesterol exchange between LDL and HDL [46], PCYOX1 (cluster 5, Fig. 5A) a pro-oxidant enzyme associated to LDL [47]and the GPI degrading enzyme GPLD1 (cluster 7, Figure 5B) [48]. Concerning platelet functionboth COVID-19 ICU and non-ICU patients show a reduction in structural proteinssuch as VCL, SPARC, PLEK, TLN1, TAGLN2, FLNA (all present in cluster 4Figure 5A) and TMSB4X (cluster 3, Fig. 5A), a possible indication of thrombocytopeniaas already reported in COVID-19 patients [49], while a reduction of secreted platelet factorssuch as PF4 and PPBPwas observed only in COVID-19 non-ICU patients. Other processes that are altered mainly in COVID-19 non-ICU patients (cluster 7, Fig. 5B) refer to endothelial cell integritysuch as VASNa factor downregulated during vessel repair [50], the vascular endothelial cadherin CDH5and the regulation of glucose metabolism by the adipose tissueincluding Adiponectin (ADIPOQ) and Retinol-Binding Protein 4 (RBP4).

Interestinglywe also detected a small set of proteins whose levels are decreased in COVID-19 non-ICU patients and increased in COVID-19 ICU/Fwhich play a role in the complement (C8G, C8B, CFB, C1QB and C1QC, cluster 12 Fig. 5C) and platelet/coagulation (SERPIN A6, SERPIN D1, F2 in cluster 12 and KNG1, PLG in cluster 6 Fig. 5C) cascadesconfirming that these processes may be differently modulated depending on disease severity.

### Validation of alterations related to neutrophil hyper-activity.

**Fig. 6:**
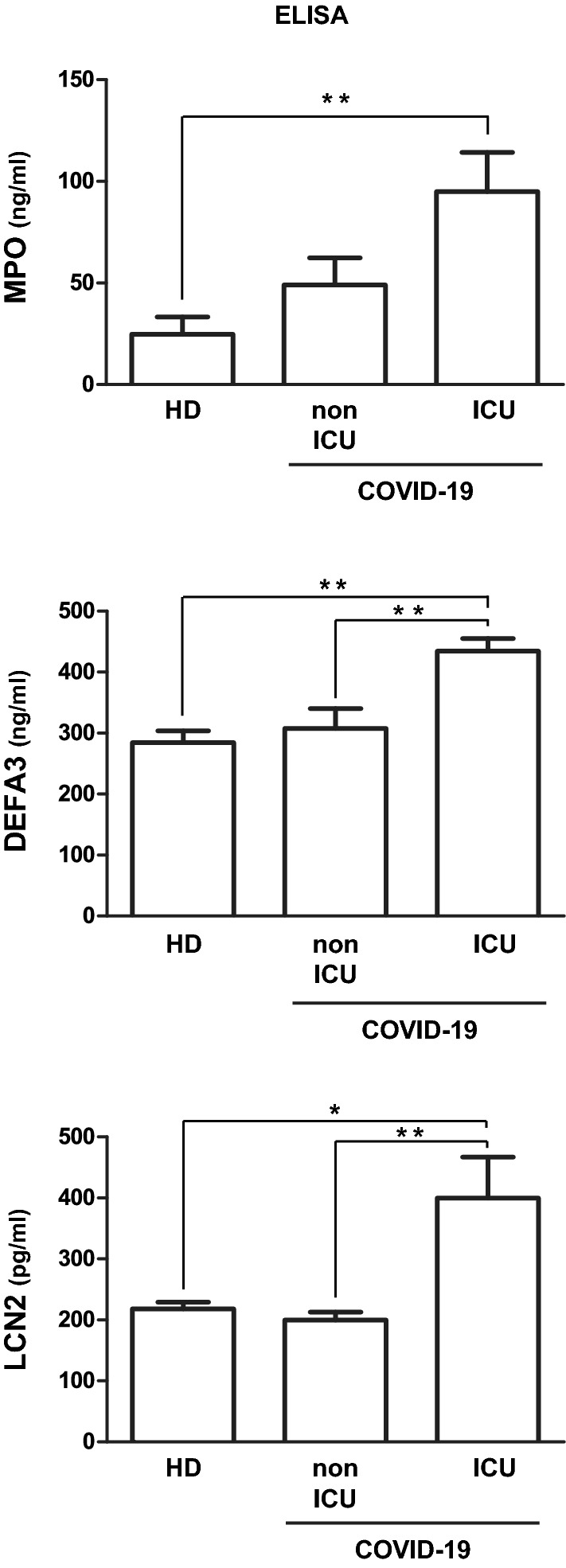
ELISA analysis of plasma levels of MPO, DEFA3 and LCN2 from 6 COVID-19 ICU patients6 COVID-19 non-ICU patients and 6 HD. Data are presented as the mean ± SD. * = P < 0.05** = P < 0.01

ELISA was used to confirm some of the changes observed in the proteomic analysisfocusing on the hyper-activation of neutrophils. For this analysiswe compared independent groups of 6 COVID-19 patients who required ICU admission and 6 COVID-19 patients who did not require it. Six healthy donors were also included in the analysis. Demographicsclinical baseline characteristicstherapy and cause of death are shown in Additional file 1: Table S1. When compared to the cohort of patients used for the proteomic analysismajor differences for COVID-19 ICU patients were the number of deceased patients (1 vs 8) and oxygen saturation median (96 vs 89,5)while for COVID-19 non-ICU patients the sample collection time after hospitalization (IQR 4.5 days vs 2.55 days). In particularwe focused on 3 neutrophil granule proteins, MPO, DEFA3 and LCN2/NGALwhose plasma levels were increased in non-ICU and ICU/F patientsat a higher extent in latter group (MPO: 1.3 vs 2.4DEFA3: 2.9 vs 3.6; LCN2/NGAL 2,1 vs 2.9). Changes in the levels of the 3 proteins discriminate between COVID-19 ICU patients and HD (Fig. 6) whileat variance with proteomic resultsno significant difference was observed between COVID-19 non-ICU patients and HD, suggesting that the variability in the levels of these proteins in the non-ICU group is higher than what estimated in the proteomic analysis. Conversely, DEFA3 and LCN2/NGAL shows significant differences between COVID-19 ICU and non-ICU patients (Fig. 6) confirming that neutrophil activation could be a hallmark of COVID-19 severity.

## Discussion

Herewe reported a proteomic analysis of plasma of COVID-19 patients with severe/fatal pneumonia compared to COVID-19 patients with pneumonia not requiring ICU admission with the aim of identifying molecular features associated to disease progression.

Our data show that several proteins belonging to the complement cascades are mainly increased in COVID-19 patients requiring ICU admissionin combination with a series of proteins playing a role in acute inflammatory responseplatelet and neutrophil function that are progressively increased in ICU/F versus non-ICU COVID-19 patients. Our results are highly consistent with the plasma/sera proteomic signatures of severe COVID-19 patients published to date [12–16, 51–61]. Noteworthysome of the identified proteins involved in the inflammatory responseplatelet and complement cascadessuch as CRP, B2M, CST3, SERPIN A3, PLG, C1R, were recently identified as predictors of future worsening of the clinical conditionand CRP, CD14, B2MSERPIN D1, C1QB, C1QC, SERPIN A3 were predictive of a longer need for inpatient treatment [62].

The complement system generates a highly regulated innate immune response mediated by both the perturbation of target membranes and the generation of a proinflammatory response through a proteolytic cascade that is initiated by three different pathways (classicalalternative and lectin) [63]. We observed an increase in the levels of several complement proteinsmost of which are exclusively induced in severe COVID-19 patientswhile upregulation of the lectin receptor MBL2 was also detected in COVID-19 non-ICU patients and further increased in COVID-19 ICU/F. InterestinglyGutmann and colleagues reported complement activation as a core component of the dysregulated immune response to COVID-19with elevated MBL2 being a predictor of 28-day ICU mortality [51]. Moreoverinduction of complement factors has been reported in both proteomic and transcriptomic studies of blood and lung samples from patients with severe COVID-19 [58, 64, 65]. Of noteseveral lines of evidence support that SARS-CoV-2 can directly activate complement through the alternative and the lectin pathwayswhich are both highly represented in our proteomic analysis [66–68]. Howeverseveral inflammatory processes can account for the indirect activation of complement cascades.

Among the different class of plasma proteins whose levels are altered in COVID-19 ICU/F patientswe have focused our interest to neutrophil markers because these cells are key players in the innate immune system [69] and an important contribution of a dysregulated activity of these cells to COVID-19 disease severity is emerging from a plethora of evidence [52, 70, 71]. In particulara strict functional interaction between neutrophils and the complement cascade has been describedwhich is bidirectional andif not tightly regulatedmay establish a self-amplifying loop which can lead to hyper-activation of the immune system and thrombotic microangiopathy [72–74]. ELISA of an independent group of patients have confirmed that MPO, LCN2 and DEFA3 are increased in COVID-19 ICU/F patients when compared to HDand observed that LCN2 and DEFA3 can discriminate ICU/F from non-ICU COVID-19 patients.

Neutrophils contribute to the innate immune response through both pathogen phagocytosisinflammation and release of NETsextracellular traps composed of neutrophil-derived chromatin and microbicidal proteins [69, 75]. Lung-recruited neutrophils are characterized by exacerbated production of IL-8IL-1βIL-6and CCL3/4 along with elevated levels of neutrophil elastase and MPO. The compartmentalization of transcriptionally active and highly inflammatory neutrophils in the lung participates in driving acute respiratory distress syndrome (ARDS) [75]. The increase in the levels of histone H4, MPO, LCN2, PGLYRP1and DEFA3 detected in our proteomic analysis strongly support the observation that excessive NET formation occurs in severe COVID-19 patients [72, 76–81]. Accordinglyhistological detection of NETs has been observed in the microvasculature of lungkidney and heart of these patients [82–84]. NETs also activate platelets and the clotting cascadeand are integral part of vascular thrombi [85, 86]. On the other handplatelet-derived and complement factors are known to trigger NET formationunderling how each process may promote the others [86, 87]. NETosisa regulated form of neutrophil deathhas been implicated in the pathogenesis of COVID-19 [88]. Opsonized pathogens may trigger NETosis through the engagement of neutrophils complement receptors and complement components are able to stabilize NETs by preventing DNase I mediated clearance [89]. On the other handneutrophils may activate the C3-convertase through proteins present on the neutrophil surface [90] and in the NETs [91]. Moreovermyeloperoxidase and neutrophil granule proteases may activate properdin and potentiate complement cascade on NETs [92]. Related to thiswe also detected an increase of VCAM and various ECM componentswhich could be indicative of vessel damageas well as red blood proteinsa possible indication of hemolysis.

### Limitations of the study

The main limit of our study is the small number of patients that were analysed. To counter this problemwe have also analysed by ELISA some of changes in the level of neutrophil granule proteins detected by proteomic analysis in an independent small cohort of patients.

A further limitation is the difference in median age of healthy donors with respect to COVID-19 patients (HD: 43non-ICU: 67.5ICU/F: 65) as well as the absence of morbiditywhich may have influenced the proteomic results independently of SARS CoV-2 infection. Age and comorbiditiessuch as obesityare well-known risk factors for severity progression in COVID-19with obesity being associated with neutrophil activation [93]. Howeverour study was mainly focused on the comparison between ICU/F and non-ICU COVID-19 patientsand the HD group was included just a control arm. The two groups of COVID-19 patients have homogeneous clinical baseline characteristicsandin this casecomorbidities should not have contributed in a relevant manner to the observed proteomic differences of plasma profiles.

## Conclusion

We have provided an in-depth analysis of proteomic changes in the plasma of COVID-19 patients in relation to the disease severity statewhich may help to elucidate the molecular basis of COVID-19 severe pathogenesis and provide a framework for studies aimed at identifying biomarkers with prognostic values for the COVID-19 outcome.

## Supplementary Information

**Additional file 1: Tables S1**: Demographics, clinical baseline characteristics, therapy and cause of death of COVID-19 patients.

**Additional file 2: Tables S2**: MaxQuant software analysis of peptides detected by mass spectrometry from plasma of COVID-19 patients.

**Additional file 3: Table S3**: MaxQuant software identification of proteins from plasma of COVID-19 patients.

**Additional file 4: Table S4**: Proteins with statistically different levels in COVID-19 patients.

**Additional file 5: Table S5**: Analysis of biological processes associated to the proteins altered in COVID-19 patients.

**Additional file 6: Table S6**: Cluster analysis of proteins modulated in the plasma of COVID-19 patients.

## Data Availability

The mass spectrometry proteomics data have been deposited to the ProteomeXchange Consortium via the PRIDE [94] partner repository with the dataset identifier PXD036491.

## Abbreviations

A1BG: Alpha-1-B glycoprotein
ADIPOQ: Adiponectin
APCS: Serum amyloid P-component
APMAP: Adipocyte plasma membrane associated *protein*
APOA2: Apolipoprotein A2
APOA4: Apolipoprotein A4
APOC1: Apolipoprotein C1
APOC3: Apolipoprotein C3
APOD: Apolipoprotein D
APOE: Apolipoprotein E
APOH: Apolipoprotein H
APOL1: Apolipoprotein L1
APOM: Apolipoprotein M
ARDS: Acute respiratory distress syndrome
B2M: Beta-2-microglobulin
CA1: Carbonic anhydrase 1
C1Q: Complement component 1q
C1QB: Complement C1q subcomponent subunit B
C1QC: Complement C1q subcomponent subunit C
C1R: Complement C1r subcomponent
C1S: Complement C1s subcomponent
C3: Complement C3
C6: Complement component C6
C9: Complement component C9
C8B: Complement component C8 beta chain
C8G: Complement component C8 gamma chain
CCL3/4: C–C motif chemokine ligand 3/4
CD14: Monocyte differentiation antigen CD14
CDH5: Cadherin-5
CETP: Cholesteryl ester transfer protein
CFB: Complement factor B
CFRH1: Complement factor H-related *protein1*
CFRH2: Complement factor H-related *protein 2*
COLEC11: Collectin-11
CRP: C-reactive *protein*
CST3: Cystatin-C
DEFA3: Neutrophil defensin 3
ECM: Extracellular matrix
EFEMP1: EGF-containing fibulin-like extracellular matrix protein 1
ELISA: Enzyme linked immunosorbent assayF10 coagulation factor X
F11: Coagulation factor XI
F2: Prothrombin
F5: Coagulation factor V
F9: Coagulation factor IX
FCN3: Ficolin 3
FGA: Fibrinogen alpha chain
FGB: Fibrinogen beta chain
FGG: Fibrinogen gamma chain
FLNA: Filamin A,
FN1: Fibronectin 1
GPLD1: Phosphatidylinositol-glycan-specific phospholipase D
GPX3: Glutathione peroxidase 3
HABP2: Hyaluronan-binding protein 2
HBA1: Hemoglobin subunit alpha
HBB: Hemoglobin subunit beta
HDL: High density lipoprotein
H4/HIST1H4H: Istone H4
HP: Haptoglobin
ICU/F: Intensive care unit/Fatal
IL-1β: Interleukin-1 beta
IL-6: Interleukin-6
IL-8: Interleukin-8
ITH3: Inter-alpha-trypsin inhibitor heavy chain H3
ITH4: Inter-alpha-trypsin inhibitor heavy chain H4
KNG1: Kininogen 1
LBP: Lipopolysaccharide-binding protein
LC–MS: Liquid chromatography with tandem mass spectrometry
LCN2/NGAL: Lipocalin 2/Neutrophil gelatinase-associated lipocalin
LDL: Low-density lipoprotein
LGALS3BP: Galectin-3-binding protein
MBL2: Mannose-binding protein C
MASP1: Mannan-binding lectin serine protease 1
MASP2: Mannan-binding lectin serine protease 2
MPO: Myeloperoxidase
MST1: Hepatocyte growth factor-like protein
MST1/MSP: Macrophage-stimulating protein
NET: Neutrophil extracellular traps
ORM1: Alpha-1-acid glycoprotein 1
ORM2: Alpha-1-acid glycoprotein 2
PCYOX1: Prenylcysteine oxidase 1
PF4: Platelet factor 4
PGLYRP1: Peptidoglycan recognition protein 1
PLEK: Pleckstrin
PLG: Plasminogen
PON1: Serum paraoxonase/arylesterase 1
PPBP/CXCL7: Platelet basic protein
PROS1: Vitamin K-dependent protein S
QSOX1: Sulfhydryl oxidase 1
RBP4: Retinol-binding protein 4
S100A8: Protein S100-A8
S100A9: Protein S100-A9
SAA1: Serum amyloid A-1 protein
SAA2: Serum amyloid A-2 protein
SAA4: Serum amyloid A-4 protein
Serpin A1: Alpha-1-antitrypsin
Serpin A3: Alpha-1-antichymotrypsin
SERPIN A10: Protein Z-dependent protease inhibitor
SERPIN A6: Corticosteroid-binding globulin
SERPIN C1: Antithrombin-III
SERPIN D1: Heparin cofactor 2
SERPIN F2: Alpha-2-antiplasmin
Serpin G1: Plasma protease C1 inhibitor
SPARC: Secreted protein acidic and rich in cysteine/osteonectin
TAGLN2: Transgelin-2
TGFBI: Transforming growth factor-beta-induced protein ig-h3
TLN1: Talin-1
TMSB4X: Thymosin beta-4
VASN: Vasorin
VCAM1: Vascular cell adhesion protein 1
VCL: Vinculin
VTN: Vitronectin
VWF: Von Willebrand factor
