# Supplementary material for: Proteomic analysis identifies a signature of disease severity in the plasma of COVID-19 pneumonia patients associated to neutrophil, platelet and complement activation
Source: Clin Proteomics. 2022 Nov 8;19:38. doi: 10.1186/s12014-022-09377-7 (PMC9641302; doi:10.1186/s12014-022-09377-7)
Supplement: Supplementary file 1 — Additional file 1: Tables S1: Demographics, clinical baseline characteristics, therapy and cause of death of COVID-19 patients. [file 12014_2022_9377_MOESM1_ESM.docx]

**Table S1.** **Demographics, clinical baseline characteristics, therapy and cause of death of COVID-19 patients**

| Variables | Covid-19 patients | | | | Healthy Donors | |
| --- | --- | --- | --- | --- | --- | --- |
|  | **non-ICU** | | **ICU/F** | |  | |
|  | **(MS) n=10** | **(ELISA) n=6** | **(MS) n=10** | **(ELISA) n=6** | **(MS) n=10** | **(ELISA) n=6** |
| *Day admitted*  *Pre-enrollment (IQR)* | 2,5 (1,25-3,75) | 4,5 (0,25-4,75) | 2,75 (2-4,75) | 2,5 (2-4,5) |  |  |
| *Sex - n. (%)* | | | | | | |
| Female | 2 (20%) | 4 (66%) | 3 (30%) | 0 (0%) | 6 (60%) | 3 (50%) |
| Male | 8 (80%) | 2 (33%) | 7 (70%) | 6 (100%) | 4 (40%) | 3 (50%) |
| *Age-year* | | | | | | |
| *Median* (IQR) | 67,5 (60-75,75) | 70 (68,5-75,25) | 65 (60-73) | 62 (45-75,25) | 40 (37,25-43) | 43 (40,75-49,75) |
| *Range* | 50-78 | 68-78 | 53-81 | 42-78 | 32-53 | 36-53 |
| *Ethnicity - n. (%)* | | | | | | |
| White | 10 (100%) | 6 (100%) | 10 (100%) | 6 (100%) | 10 (100%) | 6 (100%) |
| *Symptoms/Pneumonia – n. (%)* | | | | | | |
| Fever | 5 (50%) | 4 (66%) | 9 (90%) | 6 (100%) | 0 (0%) | 0 (0%) |
| Dyspnea | 4 (40%) | 3 (50%) | 6 (60%) | 5 (83%) | 0 (0%) | 0 (0%) |
| Pneumonia | 10 (100%) | 6 (100%) | 10 (100%) | 6 (100%) | 0 (0%) | 0 (0%) |
| *Severity index mean* | | | | | | |
| Oxygen saturation Median R.A. (IQR) | 93 (91,25-93,75) | 93 (92-98) | 89,5 (82,5-90) | 96 (92-98) |  |  |
| Respiratory rate *Median (IQR)* | 18 (15,5-30) | 18 (16-27) | 27 (26-30) | 24 (22,25-28) |  |  |
| Pressure/oxygen concentration *Median (IQR)* | 240,5 (165,5-315) | 271 (203-279) | 129 (99,75-169) | 201 (172,5-234,75) |  |  |
|  | | | | | | |
| *Assisted oxygenation – n. (%)* | | | | | | |
| Nasal cannula | 3 (30%) | 0 (0%) | 0 (0%) | 0 (0%) |  |  |
| Ventimask | 4 (40%) | 3 (50%) | 9 (90%) | 0 (0%) |  |  |
| CPAP | 3 (30%) | 1 (17%) | 4 (40%) | 0 (0%) |  |  |
| NIV | 2 (20%) | 0 (0%) | 2 (20%) | 5 (83%) |  |  |
| IOT | 0 (0%) | 0 (0%) | 8 (80%) | 2 (33%) |  |  |
|  | | | | | | |
| *Comorbidities – n./n. tot* | | | | | | |
| *Comorbidity index n/ntot* | 6/10 | 6/6 | 9/10 | 4/6 |  |  |
| Diabetes | 1 | 1 | 2 | 0 |  |  |
| Neoplasms | 1 | 1 | 1 | 0 |  |  |
| Asthma | 0 | 0 | 1 | 0 |  |  |
| Respiratory diseases | 1 | 2 | 1 | 1 |  |  |
| Kidney disease | 0 | 0 | 1 | 0 |  |  |
| Hepatic disease | 0 | 0 | 0 | 0 |  |  |
| Neurological diseases | 1 | 3 | 1 | 0 |  |  |
| Metabolic diseases | 1 | 2 | 3 | 0 |  |  |
| Tuberculosis | 0 | 0 | 0 | 0 |  |  |
| Asplenia | 0 | 0 | 0 | 0 |  |  |
| Obesity | 2 | 1 | 4 | 1 |  |  |
| HIV | 1 | 0 | 1 | 0 |  |  |
|  | | | | | | |
| *Cause of Death– n. (%)* | | | | | | |
| ARDS | 0 (0%) | 0 (0%) | 3 (30%) | 0 (0%) |  |  |
| Heart failure/ sepsis | 0 (0%) | 0 (0%) | 5 (40%) | 1 (17%) |  |  |
|  | | | | | | |
| *Therapy – n. (%)* | | | | | | |
| Antiviral therapy | 5 (50%) | 2 (33%) | 9 (90%) | 0 (0%) |  |  |
| Monoclonal antibodies (anti IL6) | 0 (0%) | 0 (0%) | 3 (30%) | 1 (17%) |  |  |
| Antibiotics / antibacterials | 6 (60%) | 2 (33%) | 9 (90%) | 3 (50%) |  |  |
| Antifungals | 0 (0%) | 0 (0%) | 5 (50%) | 0 (0%) |  |  |
| Cortisone | 3 (30%) | 3 (50%) | 4 (40%) | 3 (50%) |  |  |
| Anticoagulants | 2 (20%) | 3 (50%) | 4 (40%) | 4 (66%) |  |  |
| Statins | 1 (10%) | 3 (50%) | 0 (0%) | 0 (0%) |  |  |
| Alpha blockers | 0 (0%) | 1 (17%) | 0 (0%) | 1 (17%) |  |  |
| Beta blockers | 3 (30%) | 3 (50%) | 2 (20%) | 2 (33%) |  |  |
| ACE inhibitors | 1 (10%) | 3 (50%) | 2 (20%) | 1 (17%) |  |  |
| Calcium channel blockers | 0 (0%) | 1 (17%) | 2 (20%) | 1 (17%) |  |  |
| Diuretics | 2 (20%) | 1 (17%) | 2 (20%) | 2 (33%) |  |  |
| Metformin | 1 (10%) | 1 (17%) | 0 (0%) | 0 (0%) |  |  |
| Insulin glargine | 0 (0%) | 1 (17%) | 0 (0%) | 0 (0%) |  |  |
| Proton pump inhibitor | 5 (50%) | 3 (50%) | 3 (30%) | 4 (66%) |  |  |
| Hydroxychloroquine sulfate | 2 (20%) | 0 (0%) | 4 (40%) | 0 (0%) |  |  |

n.= number; IQR= interquartile range; R.A.: room air
